# Streptokinase reduces *Streptococcus dysgalactiae* subsp. *equisimilis* biofilm formation

**DOI:** 10.1186/s12866-024-03540-w

**Published:** 2024-09-30

**Authors:** Lea A. Tölken, Janine V. Neufend, Oddvar Oppegaard, Karen Methling, Kirsten Moll, Sylvio Redanz, Miriam M.D. Katsburg, Murtadha Q. Ali, Patience Shumba, Bernd Kreikemeyer, Steinar Skrede, Marcus Fulde, Anna Norrby-Teglund, Michael Lalk, Bård R. Kittang, Nikolai Siemens

**Affiliations:** 1 Department of Molecular Genetics and Infection Biology, University of Greifswald, Greifswald, Germany; 2 Department of Medicine, Haukeland University Hospital, Bergen, Norway; 3 Department of Clinical Science, University of Bergen, Bergen, Norway; 4 Department of Cellular Biochemistry and Metabolomics, Institute of Biochemistry, University of Greifswald, Greifswald, Germany; 5 Center for Infectious Medicine, Karolinska Institutet, Karolinska University Hospital, Huddinge, Stockholm, Sweden; 6 Department of Translational Rheumatology and Immunology, Institute of Musculoskeletal Medicine, University of Münster, Münster, Germany; 7 Center for Infection Medicine, Institute of Microbiology and Epizootics, Freie Universität Berlin, Berlin, Germany; 8 Institute for Microbiology, Virology and Hygiene, University Medicine Rostock, Rostock, Germany; 9 Haraldsplass Deaconess Hospital, Bergen, Norway

**Keywords:** *Streptococcus dysgalactiae subsp. equisimilis*, Biofilm, Streptokinase, Necrotizing soft tissue infections

## Abstract

**Background:**

*Streptococcus dysgalactiae* subspecies *equisimilis* (SDSE) is increasingly recognized as an emerging cause of invasive diseases including necrotizing soft tissue infections (NSTIs). In contrast to the closely related *Streptococcus pyogenes*, SDSE infections mainly affect older and comorbid patients. Biofilm formation has been demonstrated in soft tissue biopsies of *S. pyogenes* NSTI cases.

**Results:**

Here, we show that bacterial aggregations indicative of biofilms are also present in SDSE NSTI. Although streptokinase (Ska) activity and biofilm formation did not correlate in a diverse set of clinical SDSE isolates, addition of exogenous Ska at an early time point prevented biofilm formation for selected strains. Deletion of *ska* in SDSE S118 strain resulted in increased biofilm forming capacity. Ska-deficient mutant strain was characterized by a higher metabolic activity and consequent metabolome profiling of biofilms identified higher deposition of a wide range of metabolites as compared to the wild-type.

**Conclusions:**

Our results argue that Ska suppresses biofilm formation in SDSE independent of its original plasminogen converting activity. However, the impact of biofilms and its consequences for patient outcomes in streptococcal NSTIs remain to be elucidated.

**Supplementary Information:**

The online version contains supplementary material available at 10.1186/s12866-024-03540-w.

## Background

Necrotizing soft tissue infections (NSTIs) are rare and severe infections that encompass necrosis of any layer of the skin and soft tissue compartment. The infections are often associated with systemic toxicity and the majority of patients require intensive care and extensive surgical interventions [1]. Even with optimal treatment, the infections are associated with high morbidity and mortality rates [2, 3]. NSTIs are associated with a wide diversity of microbial agents. However, monomicrobial NSTIs are mostly caused by *Streptococcus pyogenes* [4]. During the last decades, *Streptococcus dysgalactiae* subspecies *equisimilis* (SDSE) has emerged as a significant cause of invasive infections including NSTIs [2, 4–7]. SDSE isolates mostly express carbohydrate antigens of Lancefield group C and G. However, cases reporting SDSE harboring group A antigen, which is usually expressed by *S. pyogenes*, are emerging [8–13]. Moreover, SDSE display genetic similarity to *S. pyogenes* and consequently express a wide range of the same virulence factors, including the M-protein, secreted streptolysins S and O as well as streptokinase (Ska) [14].

Ska exclusively binds human plasminogen resulting in exposure of the active site of the zymogen. The ability of this complex to cleave additional plasminogen into active plasmin enables streptococci to degrade fibrin clots as well as extracellular matrix proteins. These processes facilitate bacterial spread through the tissue [15–18]. Furthermore, Ska-plasminogen complexes contribute to bacterial survival through cleavage of antimicrobial host compounds, including histones [19] and the human antimicrobial peptide LL-37 [20]. It has been shown that invasive SDSE isolates have significantly higher Ska activity as compared to non-invasive strains [21].

Although NSTIs are acute infections characterized by a rapid progress of tissue necrosis, a recent report identified biofilms in 32% of *S. pyogenes* NSTI cases [22]. The presence of a biofilm in patient biopsies was associated with more severe tissue involvement and a pronounced inflammatory response [22]. *S. pyogenes* biofilms were found to be under the control of the global transcriptional regulators Nra or RofA, and sortase A-linked surface proteins were essential for this trait [22]. Extended in vitro analyses of *S. pyogenes* strains from the same INFECT cohort [2], revealed that *emm*1 isolates have a uniform and good biofilm forming capacity while other *emm*-types showed a greater variation [23]. Treatment of *S. pyogenes* biofilms is difficult, since biofilms have a highly decreased susceptibility to commonly used antibiotics (penicillin G, clindamycin, rifampicin), of up to 500× MIC, requiring combination therapy and the prolonged use of antibiotics [24]. However, the association between biofilm formation and clinical characteristics of NSTI patients remains elusive.

Here, we present an SDSE NSTI case with biofilm detected in the soft tissue and we explore the role of Ska in this process. We show that SDSE can form biofilms. However, Ska plays a biofilm-preventing role, which was linked to a metabolic activity of SDSE biofilms. Based on our results we propose a new function of Ska as a potential biofilm-preventing factor.

## Methods

### Bacterial strains

SDSE isolates collected from patients identified at Haukeland University Hospital, Bergen, during the period from 2003 to 2013 were used (Supplementary Tables 1 and references [5–7]). In addition, two SDSE strains, 5005 and 6007, from the INFECT cohort were used [21]. All strains were cultured in Todd Hewitt Broth (Roth) supplemented with 1.5% (w/v) yeast extract (THY, Roth) at 37 °C and 5% CO_2_. S118Δ*ska* was generated as previously described [25–27]. Primers for knockout (Table S2) were designed using the sequence of SDSE AC-2713 (NCBI database, NC_019042.1). Correct allelic replacement events were verified by PCR and sequencing.

*S. canis* strains 990, 1000,1018, 1022, 1032, 1037, 1045, 1063, 1065, 1067, and 49,926 were kindly provided by the Konsiliarlabor für beta-hämolysierende Streptokokken in der tierärztlichen Praxis und Klinik (kleine Haustiere und Pferde), Berlin, Germany.

### Whole genome sequencing and sequence analysis

DNA was extracted with GeneJET Genomic DNA Purification Kit (Thermo Fisher Scientific). Genomic libraries were prepared using Illumina DNA Prep (Illumina), followed by 150 bp paired-end sequencing on an Illumina NovaSeq6000 instrument. Reads were trimmed using Trimmomatic v0.39 [28], assembled by SPAdes v5.14 [29] and annotated with RAST v1.073 [30]. Identification of *emm*, streptokinase, and pilus island genes was performed by BLAST search in Geneious Prime v2022.2 (geneious.com). The Center for Genomic Epidemiology website (genomicepidemiology.org) was used for multilocus sequence typing (MLST) and construction of phylogenetic trees. The trees were annotated using the Interactive Tree of Life platform, iTol v6 [31]. Pilus islands, also known as Fibronectin, Collagen and T-antigen (FCT)-regions, were classified as previously reported [32]. Some isolates harbored an FCT6-region containing an additional fibronectin binding gene (*gfba*), and were denoted as FCT6b-regions.

### Streptokinase activity assay

Ska activity in bacterial supernatants was assessed using a chromogenic assay as described by Kulisek et al. [33]. Activities were related to a plasmin control. THY was used as negative control.

### Biofilm formation on polystyrene and glass surfaces

Experiments using safranin staining for quantitative measurement were performed in 96-well polystyrene plates (Greiner Bio-One). Plastic surfaces were either left uncoated or coated with fibronectin (10 µgxml^− 1^, Corning). For CLSM analyses, experiments were performed in 8-well chamber glass slides (Lab-Tec). In brief, bacterial overnight cultures were washed and diluted to an OD_600_ of 0.05 in brain-heart infusion (Roth) media supplemented with glucose (2% (w/v), Roth). The bacterial suspension was seeded into the well plate and incubated at 37 °C and 5% CO_2_. At indicated time points, bacterial biofilms were washed twice with PBS and stained with safranin (0.001% (w/v), Roth) for 30 min. After staining, biofilms were washed three times and dried. In selected experiments, the media were supplemented with 2% (v/v) human serum (Capricorn), 2 µg human plasminogen (Sigma), and natively purified SDSE streptokinase or Streptolysin O (both Sigma), which contained human serum albumin as a stabilizer, at indicated concentrations and time points. One Unit (Ska or SLO) refers to the conversion of 50% of the substrate or 50% of lysis of a 2% erythrocytes solution in 10 min by Ska or SLO, respectively. The absorbance at 492 nm was measured and defined as indicator for biofilm mass. BHI served as negative control. Biofilms on chamber slides were washed twice with PBS and prepared for confocal imaging as described below. For treatment with antimicrobials, biofilms were grown for 28 h prior to initiating the treatment for additional 44 h. Subsequently, biofilms were processed as described above.

### Human cells, culture conditions and infections

The human keratinocyte cell line N/TERT-1 was cultured in EpiLife medium (Invitrogen). Primary normal human dermal fibroblasts (NHDFs) were cultured in DMEM (Cytivia) supplemented with 10% (v/v) FCS (Invitrogen). Cells were cultured at 37 °C under a 5% CO_2_ atmosphere.

Bacterial adherence and internalization to keratinocytes and fibroblasts were quantified using an antibiotic protection assay as previously described [34]. Cells were infected with SDSE strains at a multiplicity of infection (MOI) of 10. Two hours post infection, total bacterial counts were determined by washing and lysing the cells and plating serial dilutions on blood agar plates (Oxoid). To determine internalized bacterial counts, cells were washed 2 h post infection and extracellular bacteria were killed by addition of medium containing antibiotics (100 µgxmL^− 1^ penicillin, 100 U streptomycin). 2 h post addition of antibiotics, the cells were processed as described above.

### Antimicrobial testing

1 × 10^6^ CFUxml^− 1^ of bacterial strains were exposed to different concentrations of selected antibiotics (erythromycin (Roth), penicillin G (Jenapharm), clindamycin (Sigma)) for 3 h and plated in serial dilutions on casein agar plates. LL-37 MIC determination was adapted from Hollands et al. [20]. In brief, bacteria were grown to mid-log growth phase, washed with PBS and incubated with human plasminogen (2 µgxml^− 1^, Sigma) for 1 h at 37 °C. Bacteria were washed and LL-37 (Invivogen) and/or purified streptokinase (50 IU, Sigma) were added. After 3 h incubation at 37 °C, serial dilutions were plated on blood agar plates.

### Histological analysis

Histological analysis of patient tissue included Gram (Roth) and immuno-staining and was performed as described previously [21]. For CLSM studies, biofilms were washed and fixed with ice cold acetone (Sigma) for 1 min. The following reagents were used for the staining: anti-SDSE rabbit antiserum (Davids Biotechnology), wheat germ agglutinin AF488, anti-rabbit IgG AF647, ProLong Diamond antifade mountant with DAPI (all Invitrogen). The staining was visualized using Leica Stellaris 8 CLSM and LasX software (Leica Microsystems).

### Metabolome analyses

Biofilms were cultivated as described above, supernatants were collected, shock frozen in liquid nitrogen, and stored at -80 °C until further analysis of exometabolome. Attached biofilms were washed three times with ice cold 0.9% (w/v) NaCl, scratched in ice cold 60% (v/v) Ethanol, and collected. The wells were washed once with ice-cold double distilled water, which was also collected. The samples were shock frozen in liquid nitrogen and stored at -80 °C.

Exometabolome samples were analyzed by ^1^H-NMR measurements as described previously with modifications [35]. In brief, after addition of sodium dichloroacetate as internal standard (resulting concentration in NMR samples 2 mmol/L) the samples were centrifuged using Vivacon^®^ultrafiltration spin columns (2,000 MWCO; Satorius) to remove macromolecules present in the BHI medium and to reduce background signals. The spin columns were washed with water (LC-MS grade, VWR) several times before usage to remove traces of glycerol. Samples were centrifuged for 90 min at 7,500×g and 4 °C. Filtered samples were diluted 1:5 with ultrapure water (LC-MS grade), and a sample volume of 400 µl was mixed with 200 µl of 0.2 mol/l sodium hydrogen phosphate buffer solution, containing 30% D2O (Euriso-Top) and 1.5 mmol/L 3-trimethylsilyl-(2,2,3,3-D4) 1-propionic acid, sodium salt (TSP) (Carl Roth). The Bruker AVANCE-NEO 600 NMR spectrometer equipped with a SampleJet autosampler and a 5 mm QCI cryo probe was operated by TOPSPIN 4.0.9 software (Bruker Biospin). The identification and quantification of the metabolites was done using AMIX Viewer 3.9.15 software (Bruker Biospin). Spectra were aligned by calibrating the signal of TSP to 0.0 ppm. Signals of metabolites were identified by comparison to spectra of pure compounds from an in-house library. Integrals of metabolite peaks were compared to the integral of the ERETIC signal generated by using external calibration with the ERETIC quantification tool based on PULCON for absolute quantification [36].

Extraction of biofilm associated metabolites and GC-MS analysis was performed as described previously with minor modifications [37]. In brief, samples were thawed on ice and a mixture of internal standards was added [38]. Biofilms were resuspended by shaking and vortexing and transferred into a 15 ml tube containing glass beads with 0.1 mm diameter (Satorius). Two cell disruption cycles (2 × 40 s, 6.0 m/s) were performed with a FastPrep-24 instrument (MP Biomedicals). Biofilm extracts were transferred to another tube after centrifugation (10 min; 8,000 rpm; 4 °C) and the glass beads were washed with 6 ml ultrapure water. Washing solution and extract were combined, mixed, divided into two parts, and stored at − 80 °C for lyophilization.

Dried samples were derivatized as described previously [38]. Metabolites were analyzed by scan acquisition with an Agilent 7890B GC system (Agilent Technologies). GC-MS parameters were used as follows: the injection volume of 1 µl was split 1:10. The oven program started with an initial temperature hold at 70 °C for 2 min and continued with a heating rate of 10 °C/min up to 150 °C and 20 °C/min up to 325 °C, with a hold for 7 min. After a solvent delay of 5.8 min, mass spectra were acquired within a mass range of 50 to 500 amu. All other parameters of GC and MSD were set as described before [38].

The quantification of metabolites was performed using MassHunter Quantitative Analysis B.08.00 (Agilent Technologies) by normalizing the areas of peaks to the area of peaks of internal standard compounds. Absolute concentrations of metabolites were determined using calibrations from 0.05 to 100 nmol/sample. Relative quantification of metabolites (relative amount = area metabolite/area internal standard) was done for metabolite amounts below and above the calibration range.

### Microcalorimetric assay

Microcalorimetric measurements were carried out using the CalScreener (Symcel AB, Sweden) as previously described [24]. CalWells with prepared samples (32×) and thermodynamic references (16×) were transferred to sterile titanium vials and sealed with an individual lid to 40 cNm torque. Samples were preheated and equilibrated in two steps (10 min and 20 min, respectively) before introduction into the measuring chamber; after equilibration for 1 to 2 h, the individual heat flow (µW) per insert was recorded at 42-s intervals for 72 h. Results were analyzed using calView and calData software (Symcel AB).

### SDS-PAGE and immuno-detection of Streptokinase in bacterial culture supernatants

Bacteria were grown until exponential growth phase (10 ml liquid cultures), supernatants were collected, sterile-filtered, and proteins were precipitated using ethanol (80% [v/v] end concentration) overnight at -20 °C. Samples were normalized to equal amounts of protein (15 µg) and boiled in sample buffer (100 mM Tris (pH 6.8), 2% (w/v) SDS, 10% (v/v) β-mercaptoethanol, 20% (v/v) glycerol, and 0.05% (w/v) bromophenol blue). As molecular mass marker, pre-stained protein standards (Bio-Rad) were used. The samples were separated by 12% SDS-PAGE and transferred to a PVDF membrane. The membranes were blocked with 3% (v/v) skim milk prior to primary antibody incubations. Antibody incubations were performed according to manufacturer´s guidelines. The following antibodies were used: Streptokinase polyclonal antibody (sheep Ab, Invitrogen) and secondary Rabbit anti-Sheep IgG (H + L) HRP (Invitrogen). Band intensities were analyzed using ImageJ and normalized with the total protein amount loaded on the gels.

### Quantitative reverse transcription PCR analysis (qRT-PCR)

Bacterial were growth to mid-exponential or stationary growth phases and total RNA was isolated using RiboPure RNA purification Kit (Ambion) according to manufacturer´s guidelines. cDNA synthesis was performed using the Superscript first-strand synthesis system for RT-PCR (Invitrogen). Primer sets used for the analyses are summarized in Table S2. The real-time PCR amplification was performed with iTaq Universal SYBR Green Supermix kit (Biorad) using a StepOnePlus sequence detection system (Applied Biosystems). The levels of *gyrA* transcription were used for normalization.

### Statistics

If not otherwise indicated, statistical significance of differences was determined using the 2-tailed Mann–Whitney *U* test. Multiple comparisons were done using Kruskal Wallis test with Dunn’s post-test. Correlation analyses were determined using Spearman test. Statistics were performed using GraphPad Prism version 7 (Graph-Pad software). A p-value less than 0.05 was considered significant.

## Results

### Case report and microscopic analysis of soft tissue biopsies from the site of infection

A 57-year-old female (case ID 6007) was admitted to Haukeland University Hospital due to abrupt onset of severe pain in her right lower leg. She had received blunt trauma to her ankle the preceding week, but the current symptoms debuted just two hours prior to hospitalization. Her medical history included a mastectomy for breast cancer and a lobectomy for lung cancer Stadium 1b, but no active malignant disease or current medication. The vital signs on admission revealed no fever, hemodynamic instability or respiratory distress. Inspection of the right leg showed an indistinctly demarcated pink erythema spreading proximally from the dorsal side of the foot to the mid-leg. The erythematous area was exquisitely tender, and the patient reported a Visual Analogue Scale pain intensity of 10 out of 10. Shortly after admission, the patient’s clinical condition deteriorated, and she developed hypotension and respiratory distress. A small exploratory skin incision in the right leg was performed in the emergency room, revealing thick, grey, and oedematous superficial fascia. NSTI was suspected. Empirical treatment with penicillin and clindamycin was commenced, and the patient was transferred immediately to the operating theatre. Surgical exploration confirmed the diagnosis of NSTI and extensive surgical revision was necessary, including resection of necrotic subcutaneous tissue and deep muscle fascia. Two hours after surgery, the patient developed septic shock and respiratory failure and was transferred to the intensive care unit for treatment with inotropes and mechanical ventilation. SDSE was cultured from all soft tissue samples, and confirmed susceptible to penicillin and clindamycin. Additional surgeries were performed in the following two days, after which the patient’s clinical condition gradually improved. Two weeks after admission, she underwent successful skin grafting.

**Fig. 1:**
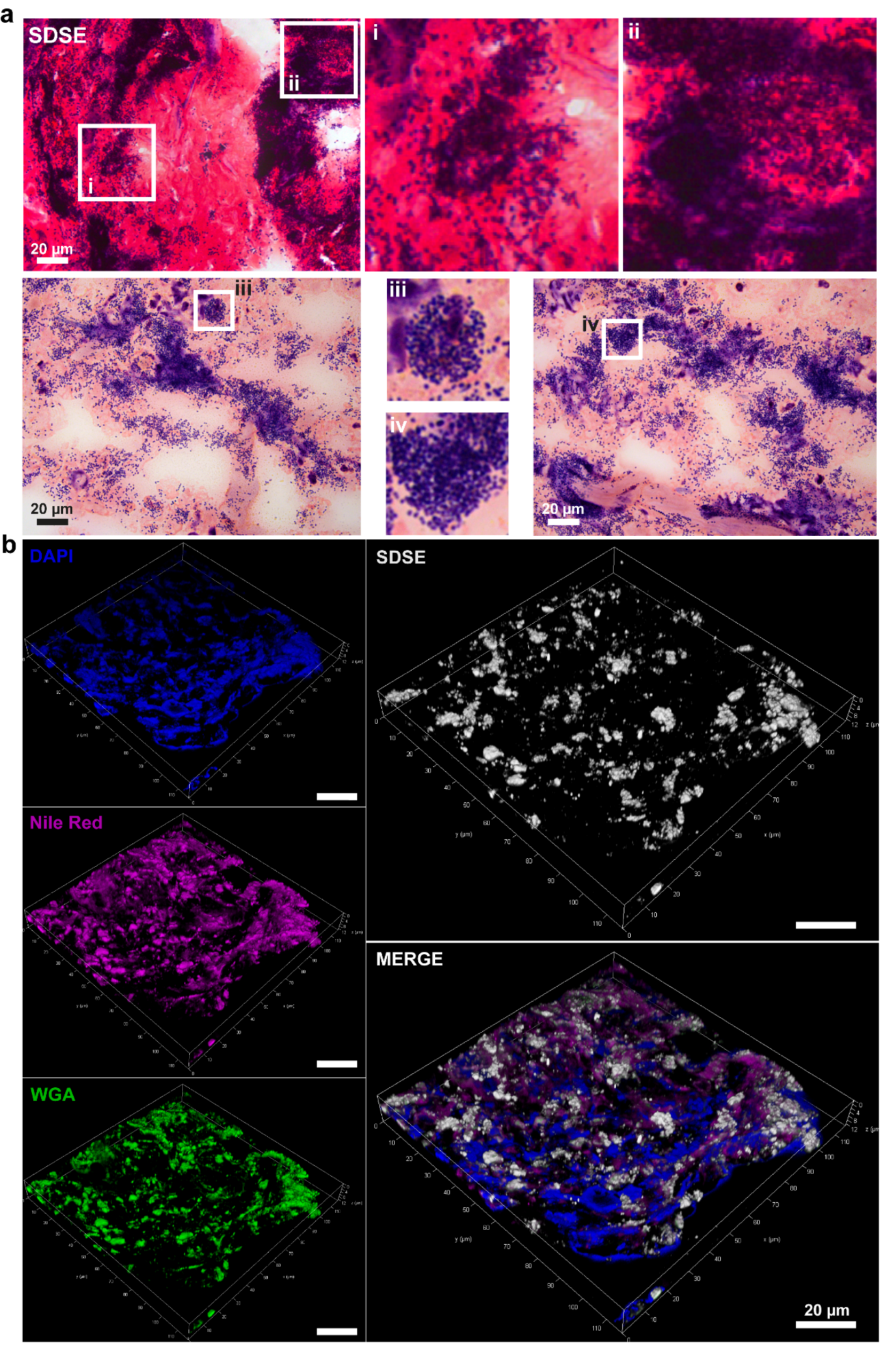
SDSE biofilm in patient biopsies. Identification of bacterial aggregates in patient biopsies by (**a**) Gram- and (**b**) immuno-staining. Representative reconstructions of CLSM micrographs visualizing biofilm. SDSE-specific antiserum, wheat germ agglutinin (WGA), DAPI, and Nile red were used

Several biopsies were collected from the site of infection, sectioned, and analyzed for bacterial presence. Gram staining revealed single cocci as well as dense bacterial aggregations consistent with biofilm communities (Fig. 1a). Next, confocal laser scan microscopy (CLSM) of immuno-stained biopsies was performed. 3D-reconstructions confirmed the multilayered nature of biofilms consisting of diffuse DNA (DAPI), lipids (Nile Red), and carbohydrates (WGA), which co-localized with the anti-SDSE staining (Fig. 1b and Supplementary Fig. 1).

### SDSE biofilm formation is independent of Ska activity

**Fig. 2:**
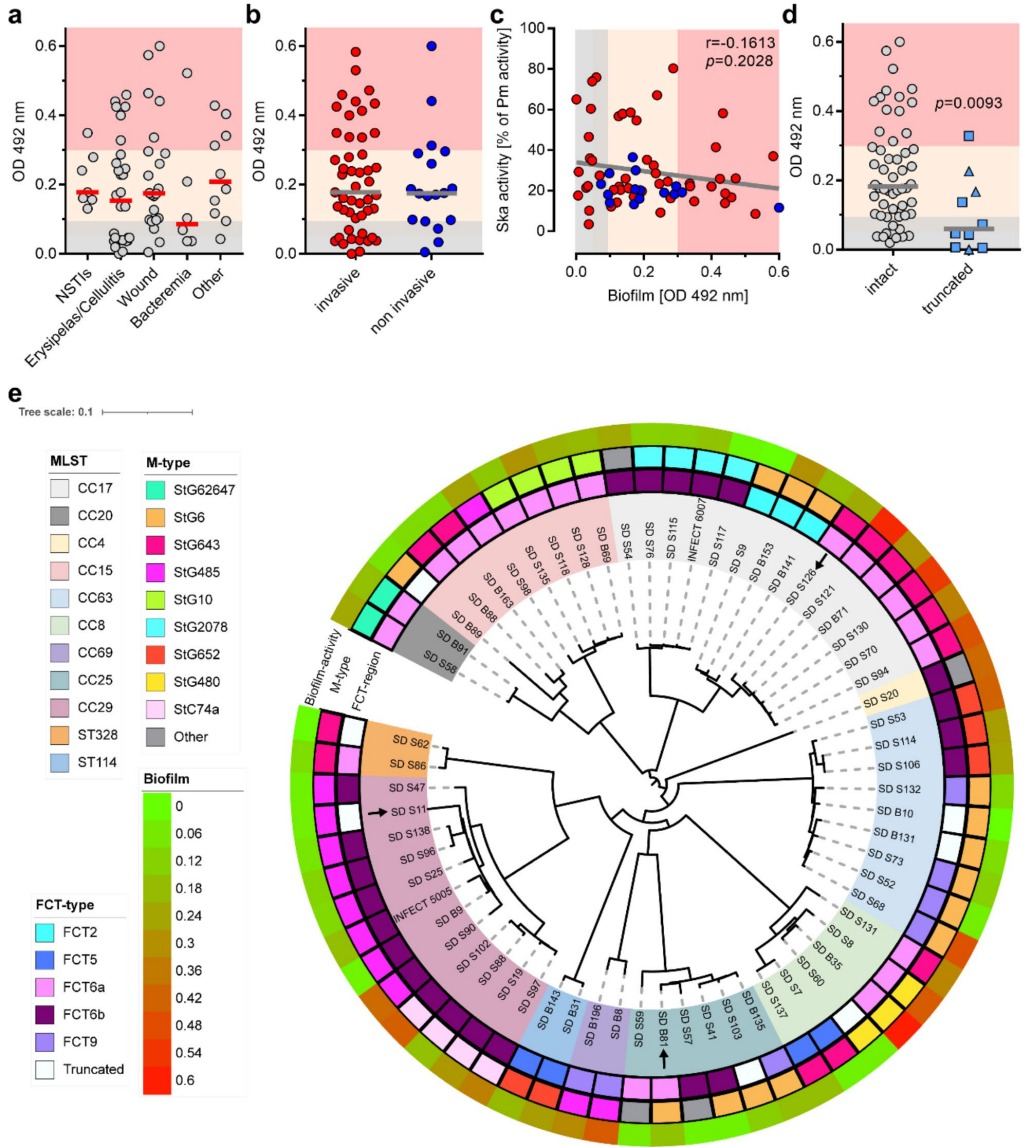
SDSE biofilm formation. Quantitative analysis of biofilm formation by 65 SDSE strains on polystyrene surfaces grouped based on (**a**) clinical presentation and (**b**) invasiveness of the infection. (**c**) Spearman correlation analysis of SDSE biofilm forming capacity and respective Ska activity of each strain. (**d**) Comparison of biofilm forming capacity between SDSE strains with intact vs. truncated *fct* (square) or *emm* (triangle) genes. (a-d) Biofilm formation on polystyrene surfaces was determined after 72 h. Ska activity in bacterial supernatants is shown as percentage of plasmin activity. Each dot represents a mean value of four independent experiments with one strain (*n* = 4). Horizontal lines depict total mean values of each group. Quartiles are indicated in colors: no or low biofilm formers (0.00-0.099, gray area); intermediate biofilm formers (0.1-0.293; yellow area), and good biofilm formers (above 0.3; red area). (**e**) Phylogenetic tree based on core genome analyses. Association of biofilm formation, sequence type (MLST), FCT- and *emm*-type of each strain are presented. Arrows indicated strains with truncated *emm-*gene. The Center for Genomic Epidemiology website (genomicepidemiology.org) was used for construction of phylogenetic trees. The trees were annotated using the Interactive Tree of Life platform, iTol v6

The observation of biofilm in the described case led us to explore whether biofilms are regularly formed by SDSE strains. Therefore, 65 previously whole-genome-sequenced SDSE strains (Supplementary Table 1) collected from invasive and non-invasive infections at Haukeland University Hospital, Bergen (Norway) from 2005 to 2013 [5–7, 21] were tested using classical biofilm readouts on polystyrene surfaces. The collection comprised diverse *emm*- as well as ST-types and the most frequent FCT/Pilus types were 6a and 6b (25 and 23, resp.; Supplementary Table S1). The majority of strains readily formed biofilms. However, the forming capacity had a wide distribution (OD_492_-values from 0.0 to 0.6; Fig. 2). Based on calculation of quartiles, isolates were classified as follows: no or low biofilm formers (OD_492_-values from 0.00 to 0.099, Fig. 2 gray area); intermediate biofilm formers (OD_492_-values from 0.1 to 0.293; Fig. 2 yellow area), and good biofilm formers (OD_492_-values above 0.3; Fig. 2 red area). Overall, no differences in biofilm formation were observed based on clinical presentation (Fig. 2a) or when comparing invasive and non-invasive strains (Fig. 2b).

Next, the strains biofilm forming capacity was related to microbiologic characteristics such as *emm*-, ST-, and FCT-types and Ska activities. No correlation between Ska activity and biofilm formation was noted (Fig. 2c). Among streptococci, particularly *S. pyogenes*, *ska* alleles can be divided in three different clusters, which exhibit differences in their respective protein activities [39, 40]. Using WGS, we did not detect correlation/clustering of biofilm formation based on *ska* gene sequence (Supplementary Fig. 2) and/or *emm*-, FCT-, and MLST-types (Fig. 2). It should be noted that SDSE *ska* sequences display only 90% homology to *ska* of *S. pyogenes* and the Ska1, Ska2a, and Ska2b clustering does not apply to SDSE (Supplementary Fig. 3). However, a small number of strains with truncated *emm* or *fct* genes was identified, and these strains showed significantly reduced biofilm forming capacity as compared to the strains with intact genetic regions (Fig. 2d-e).

### Presence of Ska prevents SDSE biofilm formation

**Fig. 3:**
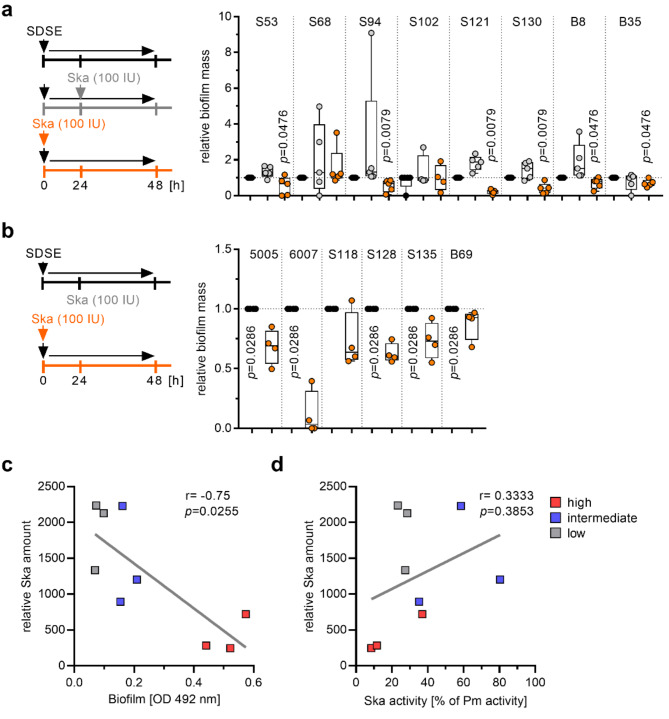
Impact of exogenous Ska supplementation on SDSE biofilms. (**a**) Comparative analysis of relative biofilm mass after addition of 100 IU exogenous Ska to high biofilm forming strains. Ska was added either directly to initial bacterial cultures (0 h) or to preformed biofilm (24 h). Each dot represents one independent experiment (*n* = 5). (**b**) Comparative analysis of relative biofilm mass after addition of 100 IU Ska to initial bacterial cultures (0 h) of intermediate biofilm forming strains. Each dot represents one independent experiment (*n* = 4). The level of significance was determined using Kolmogorov-Smirnov test. (**c-d**) Spearman correlation analyses of three randomly selected SDSE strains from indicated biofilm groups: (c) relative Ska amount vs. biofilm forming capacity and (d) relative Ska amount vs. respective Ska activity. Representative Western blot of three independent experiments is shown in Supplementary Fig. 5

As Ska treatment has been reported to have biofilm dispersing activity in *Staphylococcus aureus* infections [41, 42], we next investigated the impact of exogenous Ska supplementation on SDSE biofilm formation. Therefore, eight SDSE strains with the best biofilm forming capacity were either directly supplemented with exogenous Ska or after 24 h when the biofilm was preformed (Fig. 3a). Human plasminogen was not added to the reactions. Direct supplementation of Ska reduced biofilm formation in six out of eight strains, while addition to preformed biofilms had no effect (Fig. 3a). Next, intermediate biofilm forming strains were directly supplemented with exogenous Ska. A reduction of biofilm formation for five out of six strains was observed (Fig. 3b). To ensure that the observed effects were mediated by Ska, an unrelated, secreted, and natively purified protein, namely pore forming cytolysin SLO, was used as a control. High as well as intermediate biofilm forming strains were directly supplemented with SLO. After 48 h, no effect on biofilm formation was observed (Supplementary Fig. 4). Next, Ska secretion of nine randomly selected strains (three of each biofilm group) was analyzed via Western blot (Supplementary Fig. 5). Correlation analyses revealed that biofilm forming capacity negatively correlated with Ska amount produced by SDSE (Fig. 3c). In contrast, no correlation between secreted Ska and its activity was detected (Fig. 3d). These analyses suggest that Ska itself and not the plasminogen-mediated activity might negatively affect the biofilm forming capacity of certain SDSE strains.

**Fig. 4:**
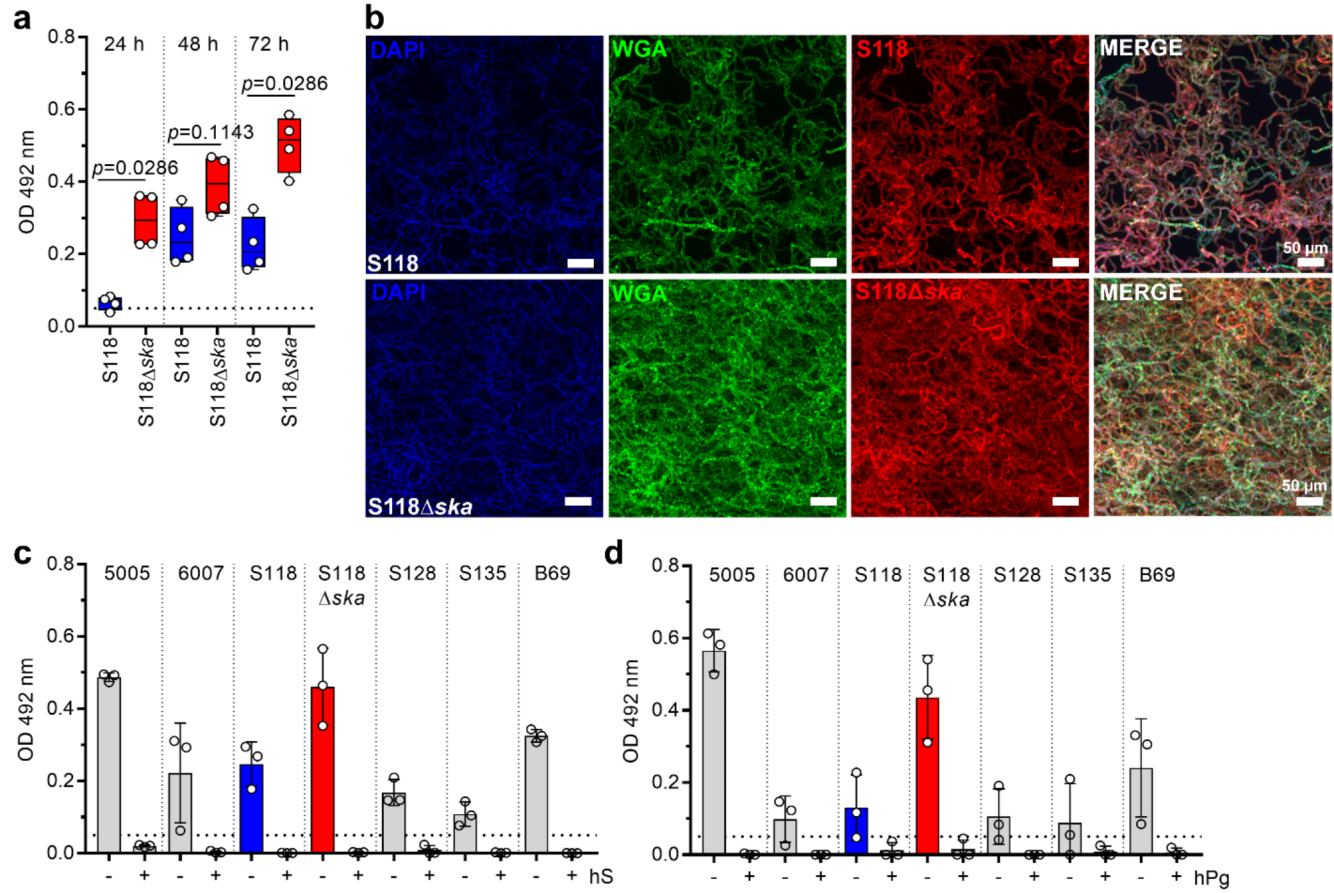
Deletion of *ska* in S118 results in enhanced biofilm forming capacity of the SDSE strain. (**a**) Quantitative analysis of biofilm formation of S118 wild-type and S118∆*ska* strains after indicated time points (*n* = 4). (**b**) Representative immunofluorescence micrographs of S118 wild-type and S118∆*ska* biofilm grown for 72 h on glass surfaces. SDSE-specific antiserum, wheat germ agglutinin (WGA), DAPI, and Nile red were used (*n* = 4). (c-d) Quantitative analysis of SDSE biofilm formation in the presence of (**c**) 2% (v/v) human serum (hS) or (**d**) 2 µg human plasminogen (hPg) (*n* = 3). Each dot in (a, c, and d) represents one independent experiment. Bars in (c-d) represents mean values ± s.d. Dotted horizontal line in (a, c, and d) depict biofilm threshold

Since the addition of Ska to the six intermediate strains had such a pronounced effect, these strains, including case strain 6007, were used for time dependent assessment of biofilm formation. Irrespective of uncoated or fibronectin-coated surfaces, all strains formed biofilms until 48 h of incubation (Supplementary Fig. 6). Several attempts to construct a *ska*-knock-out in these six strains resulted in only one successful mutant generation, namely S118Δ*ska*, which was used in subsequent experiments. In congruence with previously published data on *S. pyogenes* [19], *ska*-mutation in S118 had no impact on bacterial growth, transcription of the neighboring genes, infectivity of human keratinocytes and primary fibroblasts, or on antibiotic susceptibility (Supplementary Fig. 7a-f), but did influence LL-37 susceptibility in the presence of plasminogen (Supplementary Fig. 7g). Next, biofilm formation was monitored over a period of 72 h. In contrast to the wild-type strain, S118Δ*ska* biofilm mass steadily increased, with significantly higher values (Fig. 4a), which was further confirmed via confocal microscopy (Fig. 4b).

**Fig. 5:**
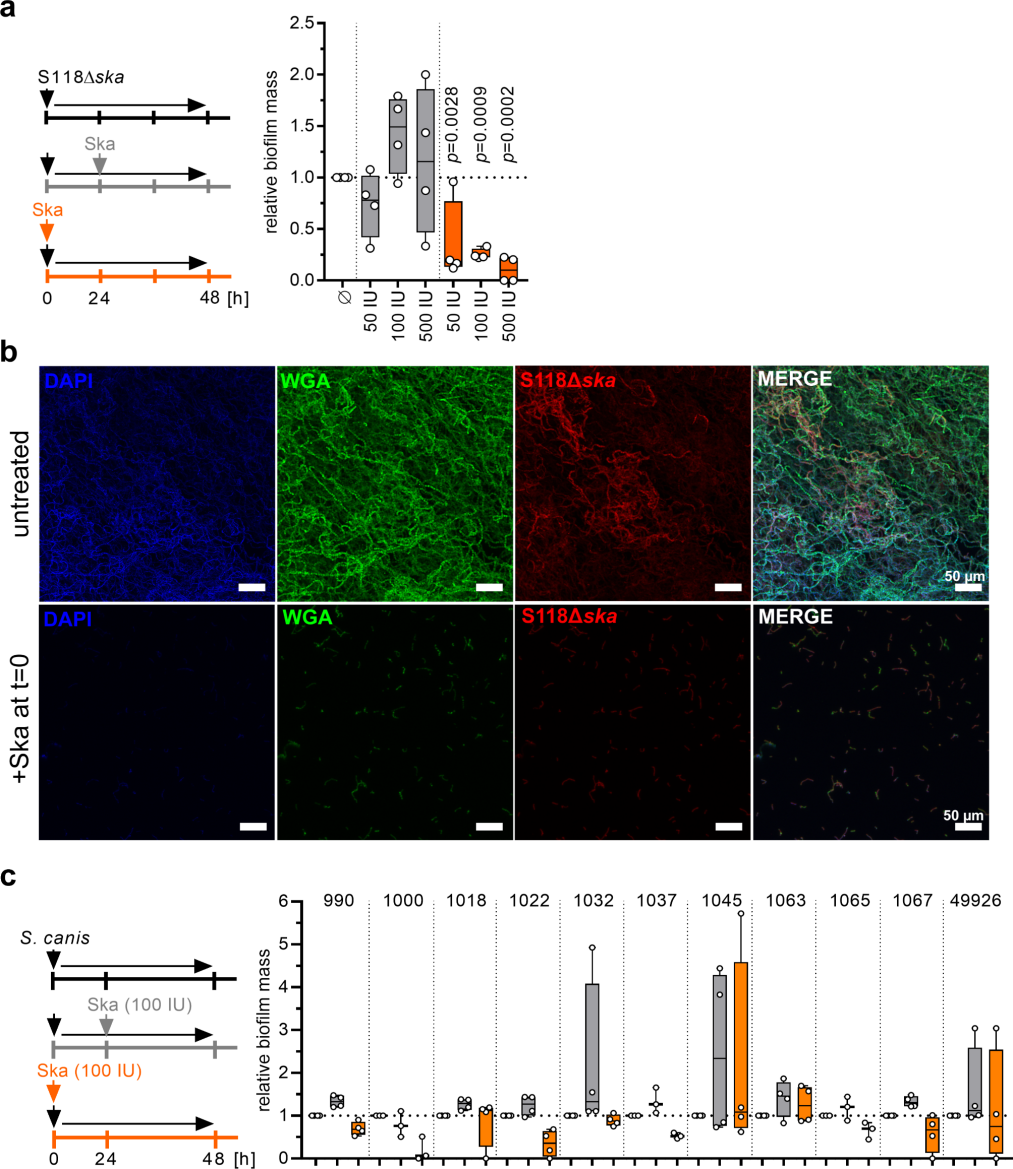
Impact of exogenous Ska supplementation on S118Δ*ska* and *Streptococcus canis* biofilms. (**a**) Comparative analysis of relative biofilm mass after addition of 50 IU, 100 IU, or 500 IU Ska to S118Δ*ska* mutant strain. Ska was added either directly to initial bacterial cultures (0 h) or to preformed biofilm (24 h). Untreated biofilms served as control (*n* = 4). (**b**) Representative immunofluorescence micrographs of untreated and 100 IU Ska treated (t = 0 h) S118∆*ska* biofilm after a total growth of 48 h on glass surfaces. SDSE-specific antiserum, wheat germ agglutinin (WGA), DAPI, and Nile red were used (*n* = 4). (**c**) Comparative analysis of *S. canis* strains relative biofilm mass after addition of 100 IU exogenous Ska. Ska was added either directly to initial bacterial cultures (0 h) or to preformed biofilm (24 h). Untreated biofilm served as controls (*n* ≥ 3). Each dot in (a and c) represents one independent experiment. The level of significance was determined using Kruskal-Wallis test with Dunn’s post correction

Since Ska is highly specific for human plasminogen, biofilm formation of strains with intermediate biofilm forming capacity was tested in presence/absence of human serum or plasminogen. Control experiments showed that addition of serum has no impact on SDSE growth (Supplementary Fig. 8). However, biofilm formation was completely abolished (Fig. 4c-d) in the presence of serum or plasminogen, even in S118Δ*ska* mutant. Next, S118Δ*ska* mutant biofilms were supplemented with exogenous Ska. Again, direct supplementation reduced biofilm formation, while addition of Ska to a preformed biofilm had no significant effect (Fig. 5a). Direct supplementation with 500 IU of Ska completely abolished biofilm formation, which was further confirmed via CLSM (Fig. 5a-b). In contrast, treatment of biofilms with up to 10×MIC of antibiotics or LL-37 did not reduce biofilm mass (Supplementary Fig. 9). As a proof-of-principle, Ska supplementation of *Streptococcus canis*, a genetically related veterinary pathogen, which does not encode *ska*, was performed. In congruence with SDSE data, Ska addition to preformed biofilms had no impact, while direct supplementation reduced biofilm mass of *S. canis* in five out of eleven strains tested (Fig. 5c).

### Increased metabolic activity and deposition of metabolites in biofilms of S118Δ *ska*

**Fig. 6:**
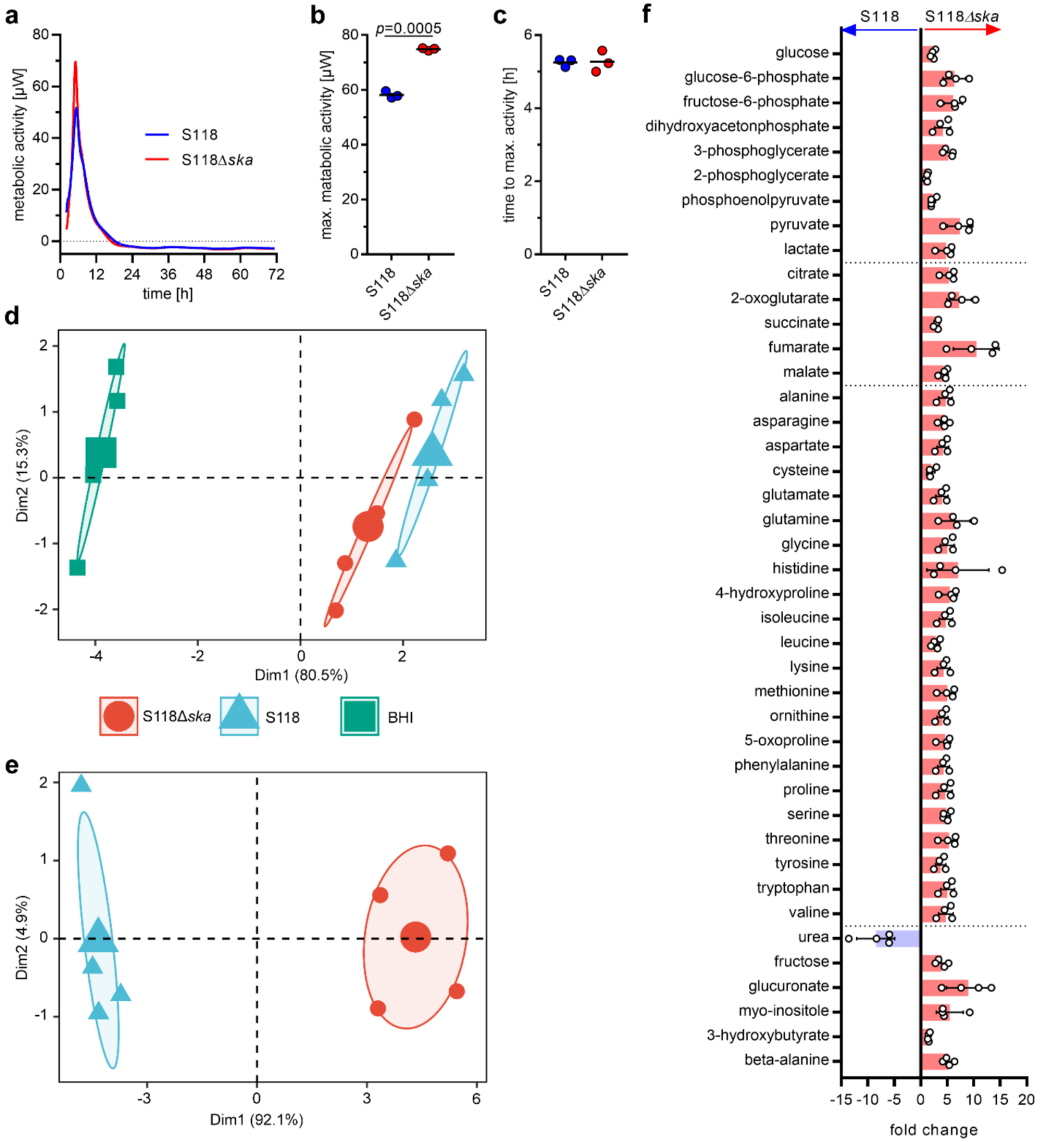
Metabolome profile of S118 wild-type and S118∆*ska* biofilms. (**a**) Exemplary curve of metabolic activity of S118 wild-type and S118∆*ska* assessed by continuous calorimetric monitoring in BHI media containing 2% (w/v) glucose. (**b**) Maximum peak of metabolic activity and (**c**) time to peak of SDSE biofilms from three independent experiments. Horizontal line depicts mean values. The level of significance was determined using student *t*-test. PCA of exometabolome (**d**) and biofilm-associated metabolites (**e**) of S118 wild-type and S118∆*ska* biofilms. Analysis of the untreated media shown in (d) was used as a control. (**f**) Fold change of biofilm-associated metabolites in S118 wild-type and S118∆*ska* biofilms quantified by GC-MS. Each dot in (d-f) represents one independent experiment (*n* = 4)

Since S118Δ*ska* continuously built up the biomass compared to the wild-type strain, we hypothesized that the mutant strain might display an enhanced metabolic activity. Therefore, continuous calorimetric metabolic monitoring of the biofilms over a period of 72 h was performed (Fig. 6a-c). The metabolic activity of both wild-type and mutant peaked at the same time point. However, the mutant was characterized by a significantly higher metabolic rate (Fig. 6b-c).

Next, metabolome profiling of the media as wells as the biofilms was performed. Analyses include the measurement of products of glycolysis/fermentation and the tricarboxylic acid (TCA) cycle as well as amino acids. No significant differences in release or consumption of metabolites between wild-type and S118Δ*ska* biofilms were noted (Fig. 6d and Supplementary Fig. 10). Both strains equally consumed glucose, trehalose, and serine. In addition, equal amounts of lactate, acetate, and ornithine were found in the media. In contrast, clear differences in metabolite composition were found within biofilms (Fig. 6e-f and Supplementary Fig. 11). Principal component analyses (PCA) showed a clear separation of biofilm-associated metabolome (Fig: 6e). Except for urea, all measured metabolites were found in higher abundance within biofilms of S118∆*ska* (Fig. 6f).

## Discussion

Biofilm formation was previously described for monomicrobial *S. pyogenes* NSTIs [22] and presents a probable cause for prolonged and/or recurrent infection, although speculative. Here, we report biofilm in tissue biopsies of a SDSE NSTI patient. Biofilm formation of SDSE clinical isolates derived from a diverse range of infections was assessed in vitro. The majority of strains readily formed biofilms. Although biofilm formation was independent of Ska activity, addition of exogenous Ska impaired biofilm formation in a majority of intermediate and high biofilm forming isolates. In addition, a *ska*-null mutant was characterized by an increased biofilm forming capacity, accompanied by higher metabolic activity as well as higher deposition of metabolites within the community as compared to the wild-type strain.

So far, Ska’s only known function is activation of human plasminogen. Ska-plasminogen complexes mediate bacterial spread through the tissues [15, 43] as well as protect streptococci from antimicrobial host compounds [19]. Ska shares a high degree of conservation with *S. aureus’* staphylokinase (Sak) with a similar mode of action [44]. However, it was recently shown that Sak activity is involved in control of *S. aureus* biofilms. High Sak-producing strains formed less biofilm as compared to non-producing strains and Sak-Pg complexes mediated dispersal of bacteria from mature biofilm [45, 46]. Recent reports also indicated that Ska itself acts as a biofilm dispersal agent against *S aureus* biofilms [41, 45]. Our analyses revealed that SDSE biofilm formation is independent of Ska activity nor it is influenced by *ska* alleles. However, direct supplementation of bacterial cultures with exogenous Ska reduced or even prevented the overall biofilm formation in a subset of SDSE strains. Furthermore, addition of human serum or plasminogen completely prevented SDSE from entering the biofilm state. This is in stark contrast to *S. aureus*, where presence of serum proteins improved its biofilm forming capacity [46]. Although speculative, plasminogen as well as serum proteins potentially prevented the sedimentation and subsequent attachment of SDSE to the polystyrene surfaces. Additional experiments with *S. canis* strains, which do not naturally encode *ska*, partly confirmed the SDSE results with reduced biofilm formation in presence of Ska. These results indicate that Ska has a general biofilm-preventing activity in streptococci.

Our results indicate that Ska is most likely involved in a multifactorial regulatory process, which controls biofilm formation. In *S. pyogenes*, biofilm formation is regulated by Nra, among others, which is known to negatively impact *ska* [22, 47] and positively regulate pili and other surface anchored/attached proteins required for initial attachment to biotic and abiotic surfaces [47–50]. During stationary growth and biofilm formation, Nra activity increases [22], which consequently results in reduced *ska* transcription and upregulation of FCT/pilus [49] structures as well as the M-protein [51, 52]. In agreement with this, whole genome sequencing (WGS) revealed that certain SDSE isolates with lower biofilm forming capacity had truncated *emm*- or FCT-genes, confirming a role of these factors in SDSE biofilm formation. However, only a limited number of strains showed such truncations, and the analyses should be expanded to allow more robust conclusions. Furthermore, CovR/S two-components system negatively regulates *ska* expression [53]. The *ska* transcripts are usually stabilized by sncRNA *fasX* [54]. It was shown that expression of CovR and *fasX* is induced upon nutrient starvation, referred to as stringent response [55]. Induction of the stringent response in late stages of bacterial growth or early stages of biofilm formation, accompanied by activation of *ska*-repressing regulators, suggests that Ska has to be suppressed during early stages of biofilm formation. In addition, the stringent response alarmone guanosine tetraphosphate and pentaphosphate ((p)ppGpp) can influence synthesis of second messenger cyclic-di-AMP (c-di-AMP) [56]. In *S. aureus*, biofilm formation was linked to increased c-di-AMP levels [57]. In general, c-di-AMP is involved in many cellular functions of Gram-positive bacteria including glutamate/glutamine regulation [58]^,^ [59] and intracellular accumulation of these amino acids induces increased synthesis of c-di-AMP [60]. In line with this, it has been shown that knockout of the c-di-AMP degrading phosphodiesterase Pde2 in *S. pyogenes* leads to increased biofilm formation [61].

It was also shown that mutation of sodium-glutamate symporter GltS leads to increased biofilm forming capacity of *S. aureus*. Due to reduced uptake of exogenous glutamate, endogenous production of glutamate/glutamine via the urea cycle was noted [62]. This result emphasized the role of urea cycle as a critical component of biofilm formation. In our experiments, high release of ornithine in biofilms of both wild-type and *ska* mutant was noted, confirming the role of the urea cycle in SDSE biofilm formation as well. In addition, urea was the only metabolite that was detected in lower abundance inside biofilms of the *ska* mutant as compared to the wild-type. In *S. pyogenes*, catabolism of arginine via generation of citrulline and ammonia is considered to mediate resistance to pH/acidic stress [63, 64]. Since the *ska* mutant showed a higher metabolic peak and higher abundance of metabolites within the biofilm, pH stress is likely encountered at a higher level as compared to the wild-type. Overall, wild-type and *ska* mutant biofilms showed similar metabolite profiles. However, the *ska* mutant biofilm was characterized by an up to 5× higher deposition of metabolites, while the biofilm mass was approximately 2.5× higher as compared to the wild-type. These findings indicate that the observed metabolic changes are partially independent of the increased biomass of the *ska* mutant biofilm and can be attributed to the lack of Ska.

## Conclusions

In conclusion, SDSE biofilm was detected in NSTI patient biopsies. Subsequent in vitro analyses of a diverse collection of isolates revealed that SDSE form biofilms, regardless of infection severity, and that Ska activity is not involved in this process. However, addition of exogenous Ska reduced SDSE biofilm formation in the majority of strains tested. Knockout of *ska* led to increased biofilm mass, higher peak metabolic activity, and increased deposition of a wide range of metabolites within biofilms. These results emphasize a new central role for Ska in SDSE biofilm formation. Nevertheless, Ska is potentially involved in a multifactorial process of biofilm regulation. The impact of Ska on metabolic regulation, biofilm formation in vivo, and consequences for patient outcome remain to be elucidated.

## Electronic supplementary material

Below is the link to the electronic supplementary material.

Supplementary Material 1

Supplementary Material 2

## Data Availability

All data associated with this study are presented in the manuscript and supplementary material. Whole genome sequencing data of SDSE strains are available at the European Nucleotide Archive. Individual accession numbers are presented in the supplementary Table 1.

## Abbreviations

^1^H-NMR: Proton nuclear magnetic resonance
AF: Alexa Flour
BHI: Brain Heart Infusion
c-di-AMP: Cyclic di AMP
CFU: Colony forming units
CLSM: Confocal laser scanning microscopy
DAPI: 4’,6-Diamidino-2-Phenylindole
DMEM: Dulbecco’s Modified Eagle Medium
FCS: Fetal calf serum
FCT: Fibronectin, Collagen and T-antigen
GC: Gas chromatography
hPg: Human Plasminogen
hS: Human Serum
IgG: Immunoglobulin G
LC: Liquid chromatography
MIC: Minimal inhibitory concentration
MLST: Multi locus sequence type
MOI: Multiplicity of infection
MS: Mass spectrometry
MSD: Mass selective detector
NaCl: Natrium Chloride
NHDFs: Normal human dermal fibroblasts
NSTIs: Necrotizing soft tissue infections
PBS: Phosphate buffered saline
PCA: Principal component analysis
(p)ppGpp: Pentaphospate
Sak: Staphylokinase
SDSE: Streptococcus dysgalactiae subsp. equisimilis
Ska: Streptokinase
sncRNA: small non coding RNA
TCA: Tricarboxylic Acid cycle
THY: Todd Hewitt Bouillon with yeast extract
TSP: 3-trimethylsilyl-(2,2,3,3-D4) 1-propionic acid, sodium salt
WGA: Wheat germ agglutine
WGS: Whole genome sequencing
