# Supplementary material for: Streptokinase reduces Streptococcus dysgalactiae subsp. equisimilis biofilm formation
Source: BMC Microbiol. 2024 Sep 30;24:378. doi: 10.1186/s12866-024-03540-w (PMC11440690; doi:10.1186/s12866-024-03540-w)
Supplement: Supplementary file 2 — Supplementary Material 2 [file 12866_2024_3540_MOESM2_ESM.pdf]

Supplementary Material

Streptokinase reduces *Streptococcus dysgalactiae* subsp. *equisimilis* biofilm formation

Lea A. Tölken[1*], Janine V. Neufend[1*], Oddvar Oppegaard[2,3], Karen Methling[4], Kirsten Moll[5], Sylvio Redanz[6], Miriam M.D. Katsburg[7], Murtadha Q. Ali[1], Patience Shumba[1], Bernd Kreikemeyer[8], Steinar Skrede[2,3], Marcus Fulde[7], Anna Norrby-Teglund[5], Michael Lalk[4], Bård R. Kittang[3,9], and Nikolai Siemens[1#]

[1]Department of Molecular Genetics and Infection Biology, University of Greifswald, Greifswald, Germany

[2]Department of Medicine, Haukeland University Hospital, Bergen, Norway.

[3]Department of Clinical Science, University of Bergen, Bergen, Norway.

[4]Department of Cellular Biochemistry and Metabolomics, Institute of Biochemistry, University of Greifswald, Greifswald, Germany.

[5]Center for Infectious Medicine, Karolinska Institutet, Karolinska University Hospital, Huddinge, Stockholm, Sweden

[6]Department of Translational Rheumatology and Immunology, Institute of Musculoskeletal Medicine, University of Münster, Münster, Germany.

[7]Center for Infection Medicine, Institute of Microbiology and Epizootics, Freie Universität Berlin, Berlin, Germany.

[8]Institute for Microbiology, Virology and Hygiene, University Medicine Rostock, Rostock, Germany.

[9]Haraldsplass Deaconess Hospital, Bergen, Norway.

*Both authors contributed equally

[#]**Correspondence:** Nikolai Siemens; **Email:** nikolai.siemens@uni-greifswald.de

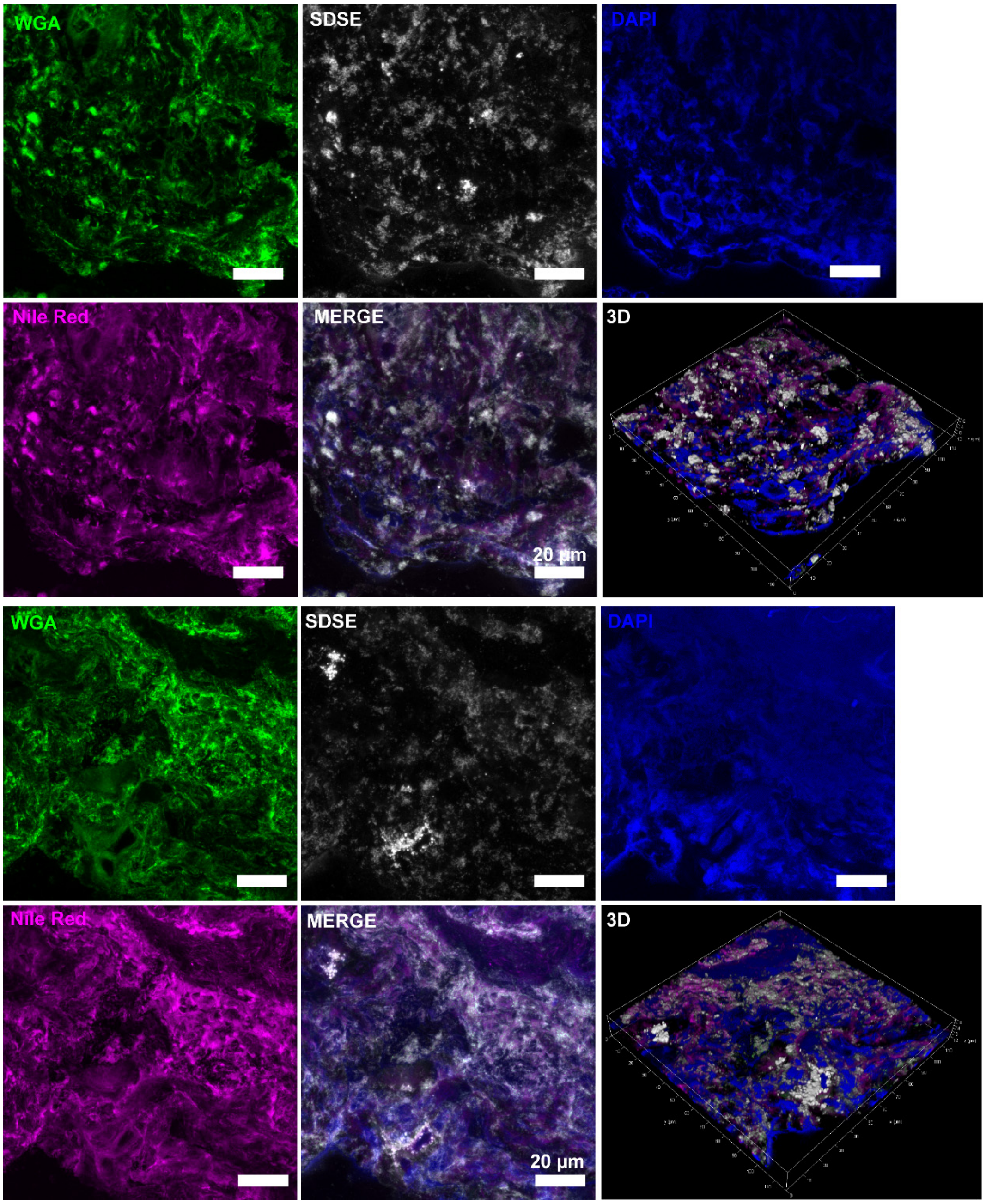

Supplementary Fig. 1. SDSE biofilm in patient biopsies. Identification of bacterial aggregations in patient biopsies by immuno-staining. Representative reconstructions of CLSM micrographs visualizing biofilm. SDSE-specific antiserum, wheat germ agglutinin (WGA), DAPI, and Nile red were used.

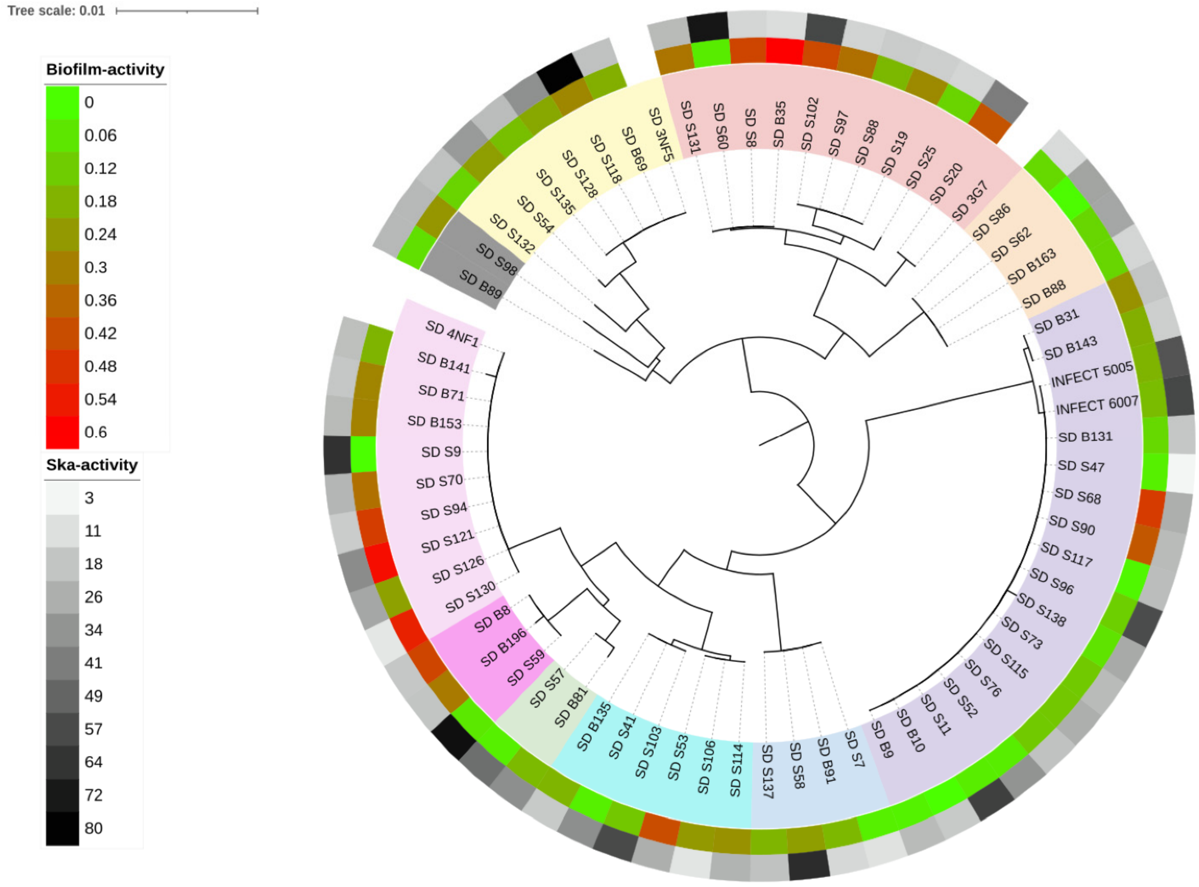

Supplementary Fig. 2. Phylogenetic tree based on *ska* alleles analysis. Association of biofilm formation and Ska activity for *ska* allele of each strain is shown. Biofilm formation (OD 492 nm) and Ska activity (% of Pm activity) are indicated as mean value of four independent experiments. The Center for Genomic Epidemiology website (genomicepidemiology.org) was used for construction of phylogenetic trees. The trees were annotated using the Interactive Tree of Life platform, iTol v6.

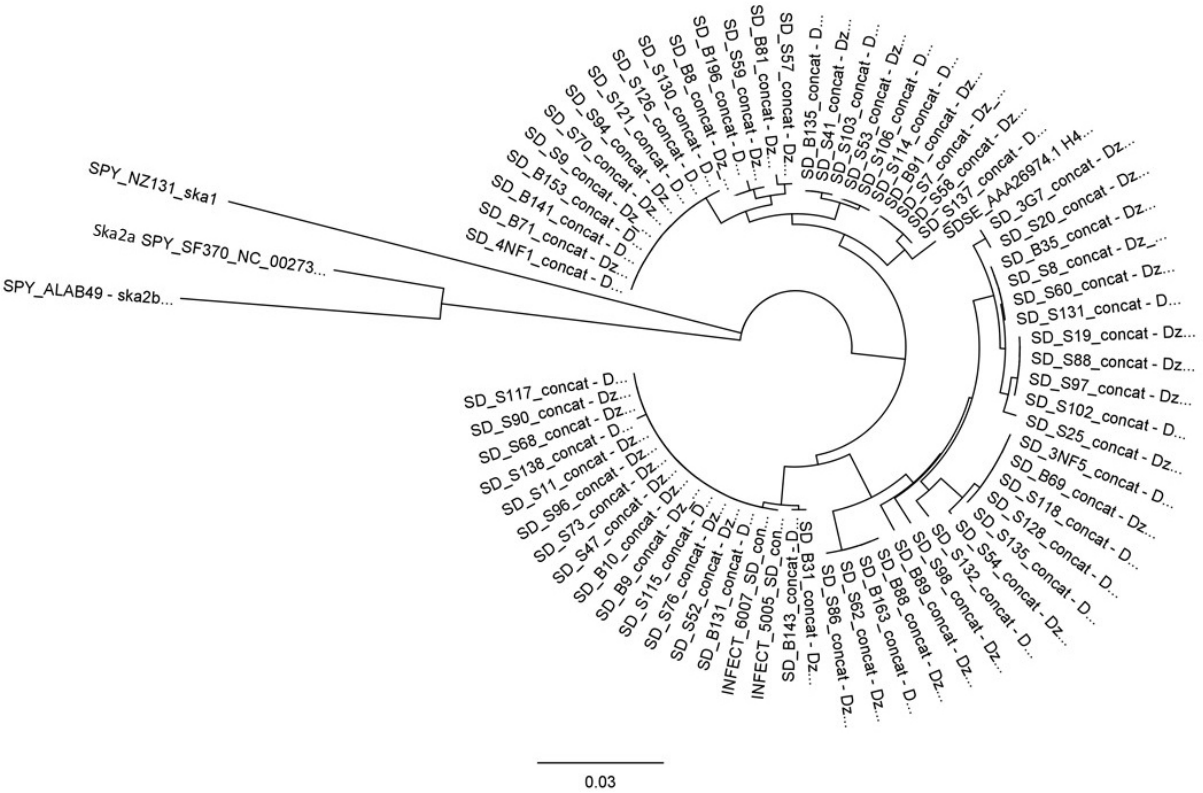

Supplementary Fig. 3. Phylogenetic tree based on *ska* alleles analysis of *S. pyogenes* and SDSE. The Center for Genomic Epidemiology website (genomicepidemiology.org) was used for construction of phylogenetic trees. The trees were annotated using the Interactive Tree of Life platform, iTol v6.

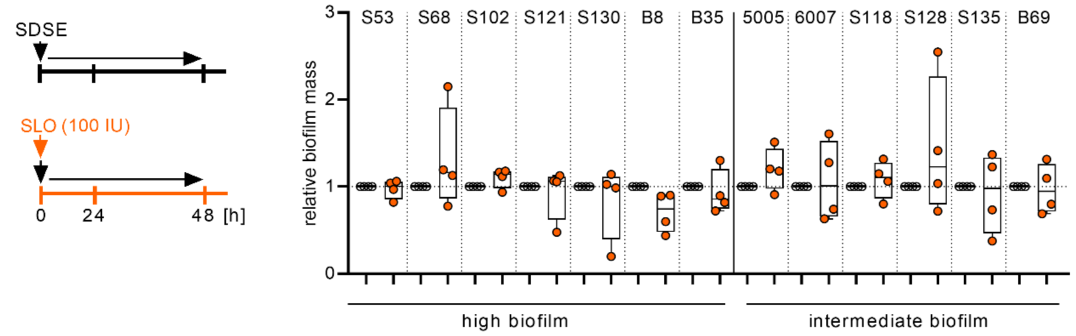

Supplementary Fig. 4. Impact of exogenous Streptolysin O (SLO) supplementation on SDSE biofilms. Comparative analysis of relative biofilm mass after addition of 100 U exogenous SLO to biofilm forming strains. SLO was added directly to initial bacterial cultures (0 h). Each dot represents one independent experiment (n=4).

a

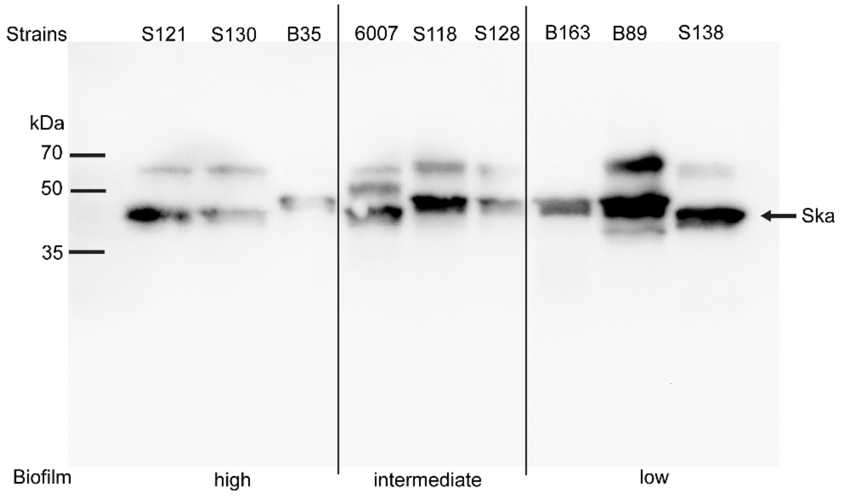

b

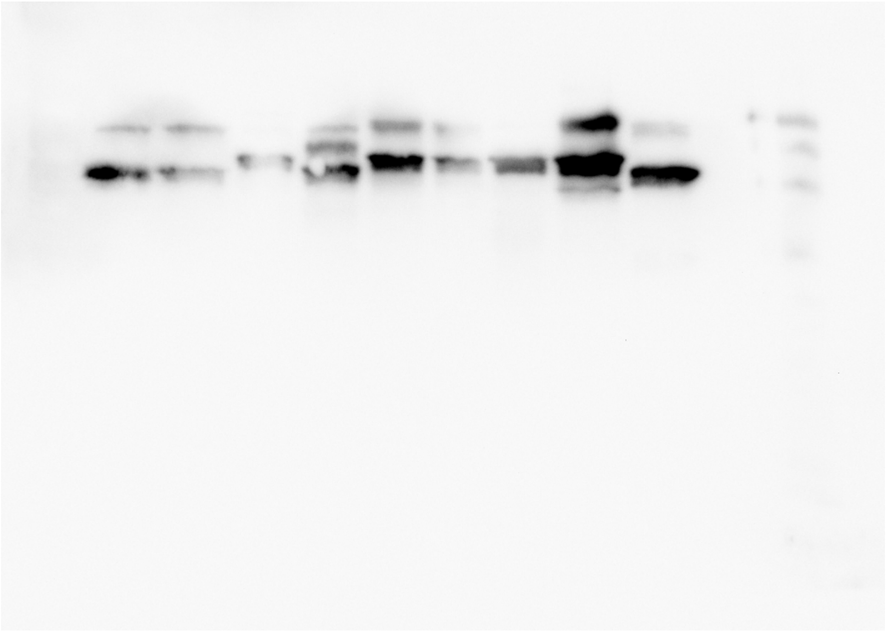

Supplementary Fig. 5. Western Blot analysis of secreted Ska. Strains were grown to the late exponential growth phase, supernatants were collected, sterile-filtered, and precipitated. 15 µg of total protein were loaded on SDS-PAGE. Sheep anti-Ska polyclonal antibody and Rabbit anti-Sheep IgG (H+L) (Invitrogen) were used for detection. (a) Representative Western Blot is shown from three independent experiments. (b) Original, unprocessed Western Blot as presented in (a) is shown. The image was acquired using Intas ECL Chemocam and 30 sec exposure time. No post-processing of the image was performed. Also, no cropping from different parts of the same gel or from different gels or fields was performed. Adobe Illustrator

CS5 was used to include labeling and lines for (a). Band intensities were analyzed using ImageJ bundled with 64-bit Java 6 and normalized with the total protein amount loaded on the gels.

The secondary antibody (RRID: AB_228457) reacts with the heavy chains of sheep IgG and with light chains common to most sheep immunoglobulins. No antibody was detected against non-immunoglobulin serum proteins. This antibody may cross-react with immunoglobulins from other species (Source: Invitrogen).

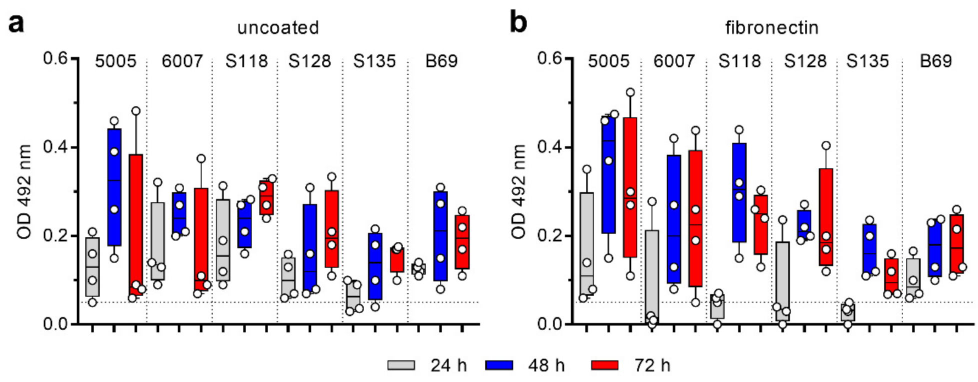

Supplementary Fig. 6. Biofilm formation kinetic of intermediate biofilm forming strains on uncoated (a) or fibronectin-coated (b) polystyrene surfaces. Biofilm formation was evaluated via safranin staining at indicated time points. Each dot represents one independent experiment (n=4).

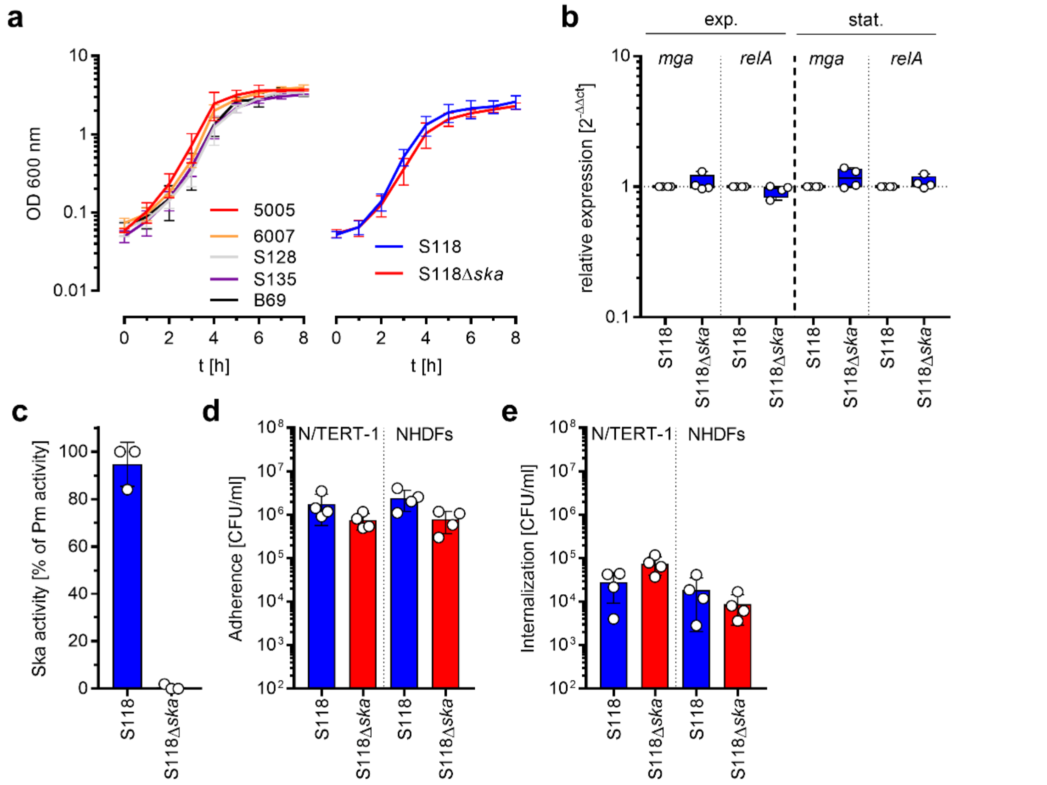

f

Strain	MIC [µg/ml]			
	Gentamicin	Erythromycin	Penicillin G	Clindamycin
5005	< 16	< 0.062	0.0156	< 0.062
6007	< 16	< 0.25	0.0156	< 0.125
S118	< 4	< 0.125	0.0156	< 0.062
S118Δ*ska*	< 4	< 0.125	0.0156	< 0.062
S128	< 4	< 0.125	0.0156	< 0.062
S135	< 4	< 0.125	0.0156	< 0.062
B69	< 4	< 0.125	0.0156	< 0.062

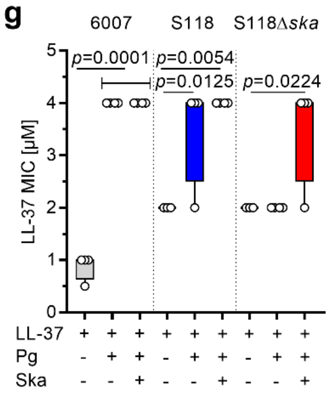

Supplementary Fig. 7. (a) Growth kinetic of intermediate biofilm forming SDSE isolates and S118Δ*ska* mutant. (b) Relative expression of genes encoding for Mga transcriptional regulator and RelA (stringent response) in S118 and S118Δ*ska* collected at indicated growth phases (exp., exponential; stat., stationary). (c) Ska activity of S118 and S118Δ*ska* assessed with bacterial supernatants derived from stationary growth phase (n=3). Keratinocytes (N/TERT-1) and human primary fibroblasts (NHDFs) were infected with S118 and S118Δ*ska* and (d) adherence and (e) internalization were assessed two and four hours post infection, resp. (n=4). (f) MICs of intermediate biofilm forming SDSE strains to commonly used antibiotics and (g) LL-37 MIC for strains 6007, S118, and S118Δ*ska* supplemented with human plasminogen (hPg) and Ska (n=4). Each dot represents one independent experiment. The level of significance was determined using Kruskal-Wallis test with Dunn's multiple correction.

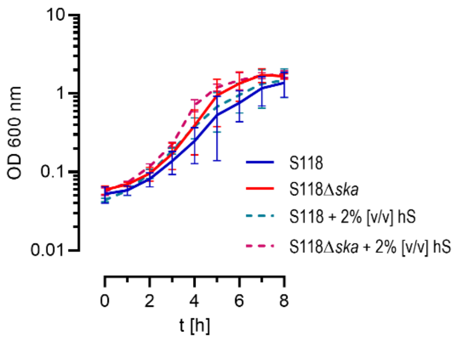

Supplementary Fig. 8. Growth kinetics of S118 and S118Δ*ska* in BHI 2% glucose with and without supplementation of 2% (v/v) human serum (hS) (n=4).

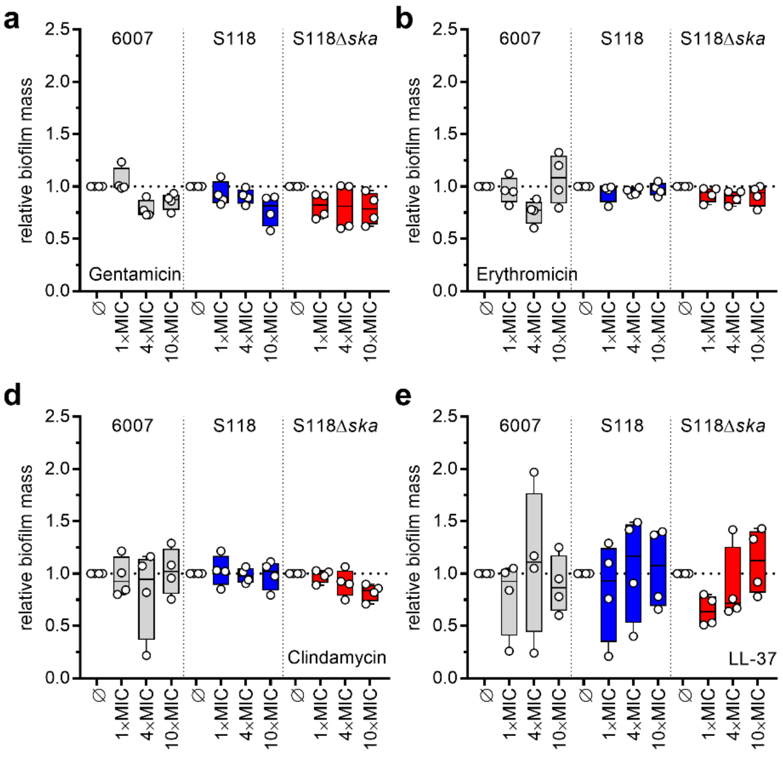

Supplementary Fig. 9. Treatment of preformed SDSE biofilm with (a) gentamicin, (b) erythromycin, (c) penicillin G, (d) clindamycin, and (e) LL-37. Antimicrobial compounds were added at indicated concentrations 24 h after inoculation. Biofilm formation was tested on polystyrene surfaces and evaluated after 48 h after addition of antimicrobial compounds by safranin staining. Each dot represents one independent experiment (n=4).

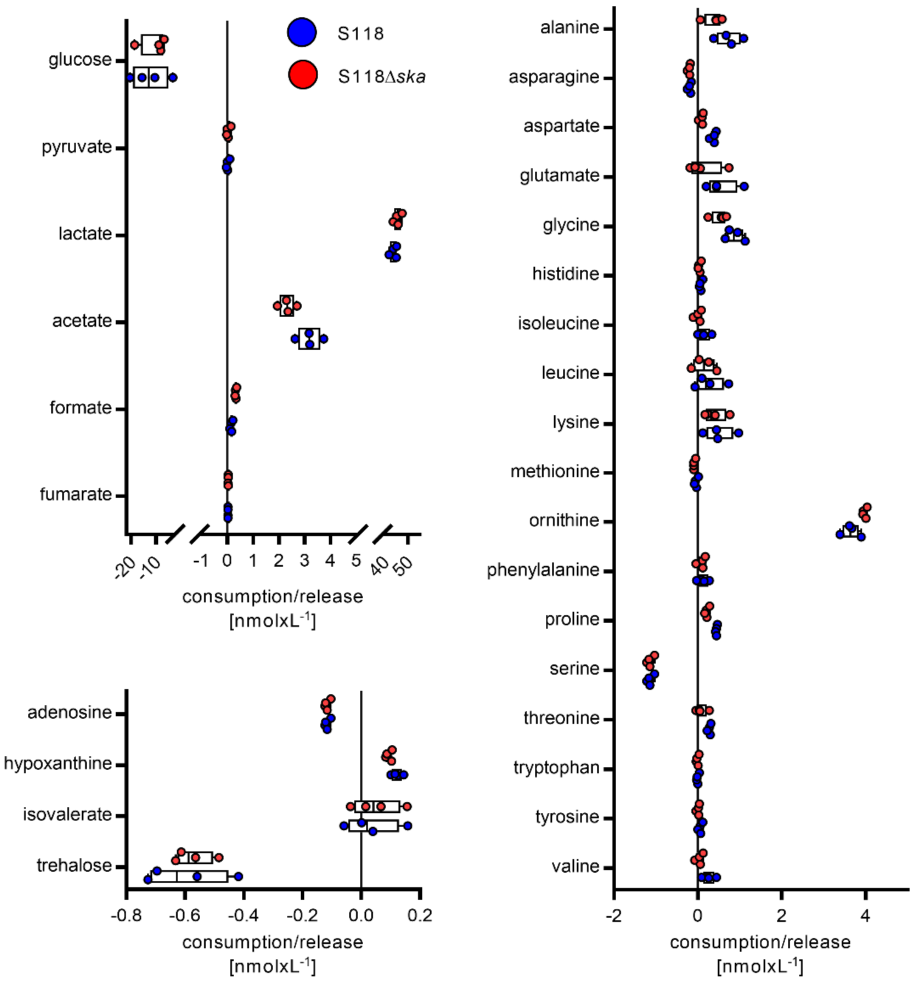

Supplementary Fig. 10. Exometabolome profile of S118 wild-type and S118Δ*ska* biofilm supernatants. Consumption/release of indicated carbohydrates, nucleotide derivates, and amino acids were determined. Metabolites were measured via ^{1}H-NMR after 72 h of biofilm formation. Each dot represents one independent experiment (n=4).

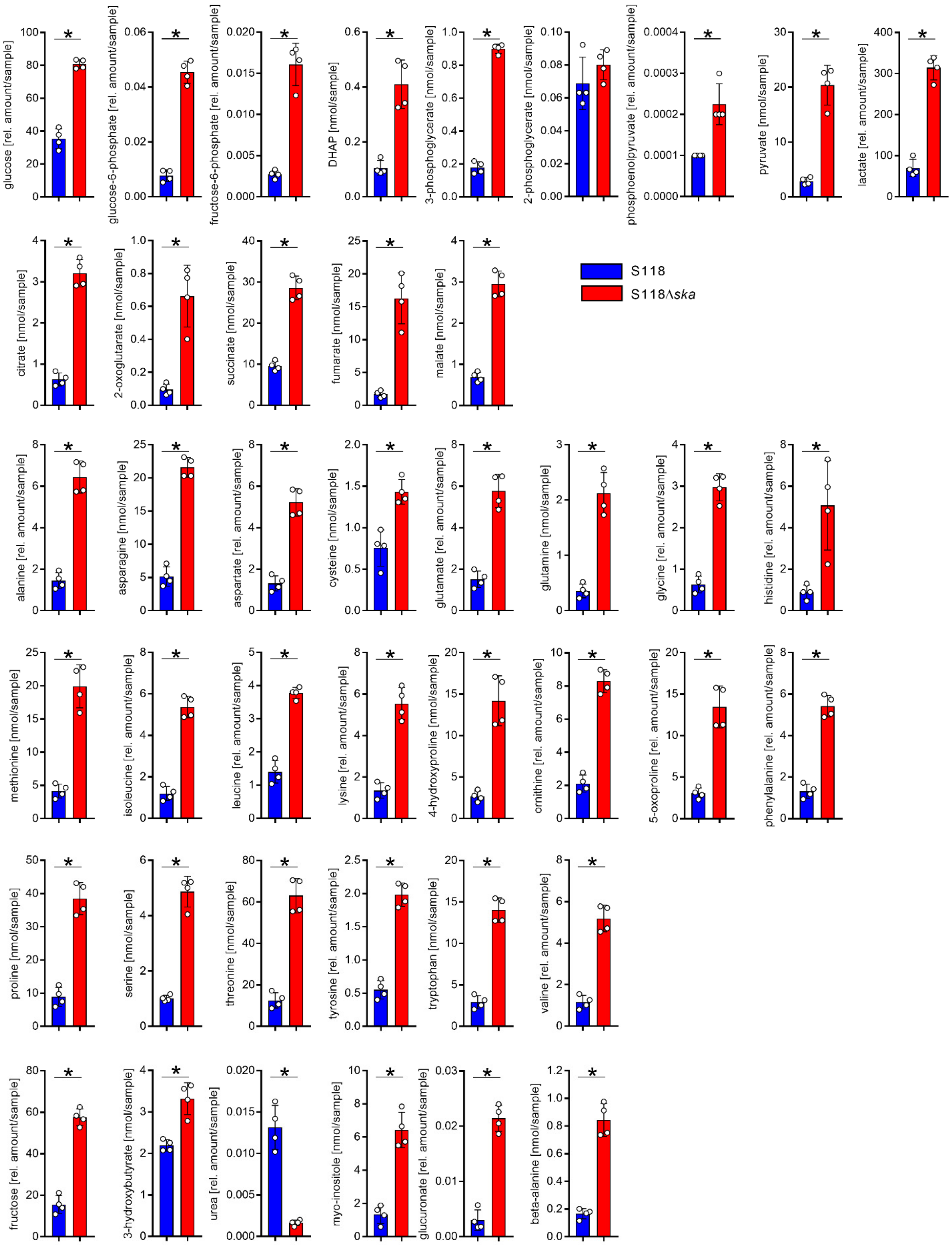

S118
S118Δ*ska*
glucose [rel. amount/sample]
glucose-6-phosphate [rel. amount/sample]
fructose-6-phosphate [rel. amount/sample]
DHAP [nmol/sample]
3-phosphoglycerate [nmol/sample]
2-phosphoglycerate [nmol/sample]
phosphoenolpyruvate [rel. amount/sample]
pyruvate [nmol/sample]
lactate [rel. amount/sample]
citrate [nmol/sample]
2-oxoglutarate [nmol/sample]
succinate [nmol/sample]
fumarate [nmol/sample]
malate [nmol/sample]
alanine [rel. amount/sample]
asparagine [nmol/sample]
aspartate [rel. amount/sample]
cysteine [nmol/sample]
glutamate [rel. amount/sample]
glutamine [nmol/sample]
glycine [rel. amount/sample]
histidine [rel. amount/sample]
methionine [nmol/sample]
isoleucine [rel. amount/sample]
leucine [rel. amount/sample]
lysine [rel. amount/sample]
4-hydroxyproline [nmol/sample]
ornithine [rel. amount/sample]
5-oxoproline [rel. amount/sample]
phenylalanine [rel. amount/sample]
proline [nmol/sample]
serine [nmol/sample]
threonine [nmol/sample]
tyrosine [rel. amount/sample]
tryptophan [nmol/sample]
valine [rel. amount/sample]
fructose [rel. amount/sample]
3-hydroxybutyrate [nmol/sample]
urea [rel. amount/sample]
myo-inositole [nmol/sample]
glucuronate [rel. amount/sample]
beta-alanine [nmol/sample]

Supplementary Fig. 11. Biofilm associated metabolites of S118 wild-type and S118Δ*ska* biofilm. Concentrations or relative amounts of individual metabolites measured in SDSE biofilm. Biofilm formation was tested on polystyrene surfaces for 72 h. Each dot represents one independent experiment (n=4). The level of significance was determined using Mann-Whitney *U*-test (*, $p<0.05$).
